# Gliotoxin and related metabolites as zinc chelators: implications and exploitation to overcome antimicrobial resistance

**DOI:** 10.1042/EBC20220222

**Published:** 2023-09-13

**Authors:** Shane G. Downes, Sean Doyle, Gary W. Jones, Rebecca A. Owens

**Affiliations:** 1 Department of Biology, Maynooth University, Maynooth, Co. Kildare, Ireland; 2 Centre for Biomedical Science Research, School of Health, Leeds Beckett University, Leeds LS1 3HE, U.K.

**Keywords:** AMR, antimicrobial resistance, gliotoxin, Nutritional immunity, quantitiative proteomics, zinc

## Abstract

Antimicrobial resistance (AMR) is a major global problem and threat to humanity. The search for new antibiotics is directed towards targeting of novel microbial systems and enzymes, as well as augmenting the activity of pre-existing antimicrobials. Sulphur-containing metabolites (e.g., auranofin and bacterial dithiolopyrrolones [e.g., holomycin]) and Zn^2+^-chelating ionophores (PBT2) have emerged as important antimicrobial classes. The sulphur-containing, non-ribosomal peptide gliotoxin, biosynthesised by *Aspergillus fumigatus* and other fungi exhibits potent antimicrobial activity, especially in the dithiol form (dithiol gliotoxin; DTG). Specifically, it has been revealed that deletion of the enzymes gliotoxin oxidoreductase GliT, *bis*-thiomethyltransferase GtmA or the transporter GliA dramatically sensitise *A. fumigatus* to gliotoxin presence. Indeed, the double deletion strain *A. fumigatus* Δ*gliT*Δ*gtmA* is especially sensitive to gliotoxin-mediated growth inhibition, which can be reversed by Zn^2+^ presence. Moreover, DTG is a Zn^2+^ chelator which can eject zinc from enzymes and inhibit activity. Although multiple studies have demonstrated the potent antibacterial effect of gliotoxin, no mechanistic details are available. Interestingly, reduced holomycin can inhibit metallo-β-lactamases. Since holomycin and gliotoxin can chelate Zn^2+^, resulting in metalloenzyme inhibition, we propose that this metal-chelating characteristic of these metabolites requires immediate investigation to identify new antibacterial drug targets or to augment the activity of existing antimicrobials. Given that (i) gliotoxin has been shown *in vitro* to significantly enhance vancomycin activity against *Staphylococcus aureus*, and (ii) that it has been independently proposed as an ideal probe to dissect the central ‘Integrator’ role of Zn^2+^ in bacteria – we contend such studies are immediately undertaken to help address AMR.

## Introduction

### Antimicrobial resistance

Antimicrobial resistance (AMR) has been identified by multiple international agencies and governments as one of the major risks to human health in the next 50 years, and beyond [1]. Moreover, it is estimated that the global economic loss associated with AMR, over the next 40 years, could be as high as $3 trillion [2]. The problem of AMR is twofold: (i) bacteria have acquired resistance to conventional antibiotics (e.g., carbapenems, aminoglycosides, fluoroquinolones and vancomycin) and (ii) there is a shortage of novel and effective antibiotics, and new targets – although this is slowly beginning to be addressed [3]. Methicillin-resistant *Staphylococcus aureus* (MRSA) accounts for up to 70% of *S. aureus* infections in clinical settings, is responsible for most invasive infections caused by AMR bacteria and is associated with a 14% fatality rate [4,5]. Vancomycin-resistant enterococci (VRE) consist mainly of *Enterococcus faecium* and *faecalis*, and vancomycin is considered the antibiotic of last resort. VRE have become common in many hospitals throughout the world and, when established, cannot be eradicated [6]. Indeed, most severe VRE infections require combination therapy because when used alone, most antimicrobial agents only effect bacteriostasis and do not clear infections. *E. faecium* along with *S. aureus*, *Klebsiella pneumoniae*, *Acinetobacter baumannii, Pseudomonas aeruginosa* and *Enterobacter spp*. comprise the priority so-called ‘*ESKAPE*’ group of bacteria. They can all cause life-threatening nosocomial infections amongst critically ill and immunocompromised individuals and exhibit potential drug resistance mechanisms [7]. It is therefore essential that new strategies are considered and deployed to overcome AMR [8].

### New directions to overcome AMR

#### Emerging antimicrobials

**Figure 1:**
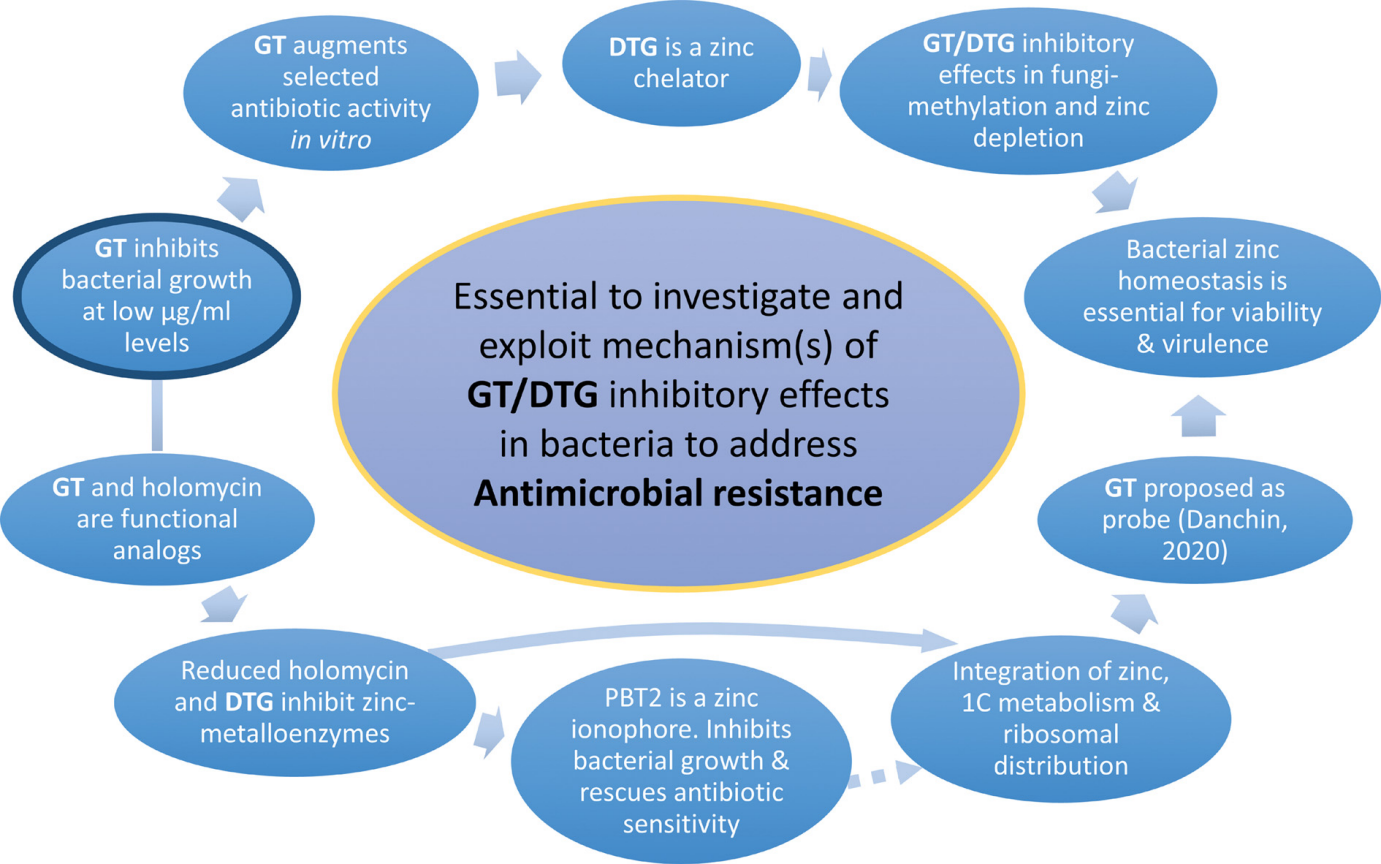
Literature-based rationale to investigate the inhibitory effects of gliotoxin/dithiol gliotoxin (GT/DTG) against bacteria to help overcome antimicrobial resistance GT/DTG inhibit bacterial growth and can also augment antibiotic activity. DTG, of fungal origin and with antifungal activity, is a zinc chelator and bacterial zinc homeostasis has been shown to be essential for growth and virulence. GT and holomycin are functional analogues, reduced holomycin and DTG inhibit specific zinc-metalloprotein activity and zinc ionophores exhibit antimicrobial activity and rescue antibiotic sensitivity. GT has independently been proposed as a probe for integrated bacterial zinc homeostasis systems with one carbon (1C) metabolism and ribosomal distribution [ 80].

The FDA-approved organogold, a *sulphur*-containing drug auranofin, has been shown to be a potent inhibitor of bacterial thioredoxin reductase TrxR, and therefore disrupts the redox balance in *Mycobacterium tuberculosis* [9]. Auranofin causes bacterial cell death, and has shown promise in *in vivo* efficacy studies in a murine model of systemic MRSA infection. Thus, it is an emerging candidate for antibacterial therapy which disrupts redox control [9]. Another class of thiol-containing metabolites, the bacterial dithiolopyrrolones (DTPs) (e.g., holomycin, thiolutin and thiomarinol B) are effective antimicrobial agents – yet their mode of action, apart from recently identified metal chelation properties, remains very poorly understood [10,11]. The hydroxyquinoline ionophore PBT2 enables zinc transport across biological membranes which results in altered intracellular zinc homeostasis [12]. Although PBT2 was abandoned as a therapeutic for Alzheimer’s and Huntington’s diseases, it showed a good safety profile. Relevantly, in 2018 it was shown that PBT2-Zn destabilizes key cellular homeostasis pathways involved in bacterial antibiotic resistance mechanisms and *in vivo* dramatically re-sensitised VRE to vancomycin, as well as enhancing erythromycin and methicillin sensitivity in other species [12]. This elegant work has clearly indicated disrupted zinc homeostasis as a new direction in overcoming AMR. Indeed, it was subsequently shown [13] that PBT2 is a Zn^2+^/H^+^ ionophore which exhibits bactericidal activity in *Streptococcus uberis* through intracellular zinc toxicity, resulting in reactive oxygen species (ROS) accumulation and dysregulated Mn^2+^ homeostasis, ultimately causing bacterial hypersensitivity to oxidative stress. PBT2 has also been proposed as a potential therapeutic to treat infection with the biofilm-forming, Gram-negative anaerobic pathogen, *Fusobacterium nucleatum* [14] and it has also been shown to rescue tetracycline-class antibiotics in multi-drug resistant *A. baumannii* [15]. However, in addition to possible neurological interactions, PBT2 is not naturally occurring and is only available by chemical synthesis. This underpins the exploration of alternative zinc-chelating compounds of microbial origin, like the epipolythiodioxopiperazine (ETP) gliotoxin (GT) or derivatives to (i) disrupt intracellular zinc homeostasis, (ii) enhance ionophore-induced zinc and ROS dysregulation, (iii) rescue antibiotic sensitivity and (iv) identify new cellular systems as antimicrobial targets [16] (Figure 1). Furthermore, like holomycin, the redox-active fungal metabolite, GT, has significant antimicrobial activity, possibly due to the zinc-chelating properties of the reduced form of GT [17–20]. Although GT is likely unsuitable for direct usage as an antibacterial due to cytotoxicity towards animal cells, it has significant potential as a ‘Pathfinder’ to identify susceptible molecular targets and potentially developing completely novel approaches to overcoming AMR in bacteria (Figure 1).

#### GT biosynthesis, self-resistance and systems interactions in fungi

**Figure 2:**
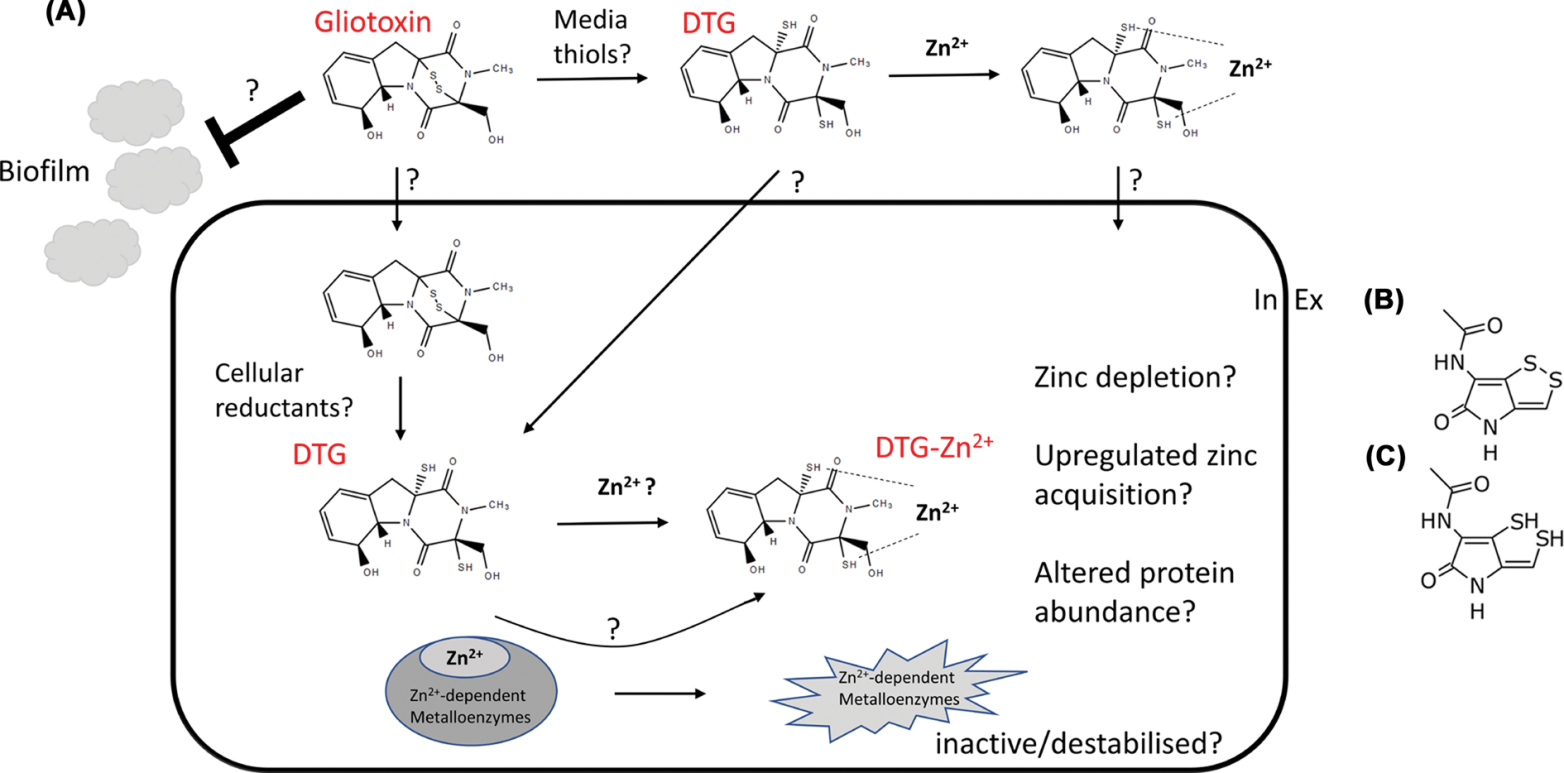
Hypothetical model for gliotoxin/dithiol gliotoxin (GT/DTG) inhibition of bacterial growth (**A**) Currently, it is unclear if GT or DTG-zinc chelate (DTG:Zn^2+^) is acquired by bacteria, and if other metals ions are capable of chelate formation with DTG. However, it is postulated that upon uptake, GT is reduced to DTG by cellular reductants (e.g., L-Cys, GSH, coenzyme A, etc.) followed by zinc chelation. Consequently, this may deplete intracellular zinc, disrupt intracellular zinc homeostasis or destabilise zinc metalloenzymes leading to growth inhibition. Altered intracellular proteome or increased abundance of zinc uptake systems may ensue, as has been observed in fungi [22]. GT addition to bacteria may also interfere with biofilm formation [51]. (**B**) Holomycin. (**C**) Reduced holomycin.

GT is a disulphide bridge-containing ETP, produced by *Aspergillus fumigatus* under zinc-limiting conditions, which can exist in oxidised, reduced and *bis*-thiomethylated (BmGT; bisdethiobis(methylthio)gliotoxin) forms [21–24] (Figure 2A). Interestingly, the non-ribosomal peptide GT can induce its own biosynthesis in *A. fumigatus* [25,26], and its ability to chelate zinc is reminiscent of siderophore-mediated iron chelation [27]. Significant advances have been made in our understanding of GT biosynthesis, including identification of the GT-encoding *gli* biosynthetic gene cluster which comprises 13 genes in *A. fumigatus*, GliZ transcription factor function, GliP non-ribosomal peptide synthetase characterization, GliG-mediated use of glutathione (GSH) as the sulphur source for biosynthetic intermediate sulphurization, GliT oxidoreductase (along with GliA an MFS transporter) as a self-resistance mechanism against GT, and GtmA (also termed TmtA) *bis*-thiomethyltransferase identification [28–35]. The latter enzyme is non-cluster encoded and dissipates GT biosynthesis [34]. These discoveries have, in turn, informed on the biosynthetic mechanism of related ETP structures in other fungi (reviewed in [23]), and holomycin in bacteria [36] (Figure 2B). Reciprocally, holomycin studies have informed advances in GT biosynthesis [37], as described below.

Moreover, three important implications derive from the aforementioned, and related studies, on GT and other ETPs. Firstly, in fungi and animal cells, intracellular GSH can convert GT to reduced or dithiol gliotoxin (DTG) which may in turn (i) disrupt cellular redox homeostasis and (ii) chelate free zinc or eject zinc from metalloenzymes [17,21,38,39] (Figure 2A). Secondly, GliT is essential to convert DTG to GT which is then effluxed from mycelia via GliA, or GtmA *bis*-thiomethylates DTG to BmGT to switch off GT biosynthesis [34,40,41]. Thirdly, DTG *bis*-thiomethylation requires 2 mol *S*-adenosyl methionine (SAM) per mol BmGT formed, in addition to forming 2 mol *S*-adenosyl homocysteine (SAH), and tethers the dissipation of GT biosynthesis to the fungal methyl-methionine cycle [41]. Capable of expression independently of the *gli* cluster, GliT activity prevents GtmA-mediated dysregulation of the cellular methyl-methionine cycle in *A. fumigatus* [33,41]. The absence of both *gliT* and *gtmA* hypersensitises the organism to exogenous GT, which can be reversed by zinc addition [42]. Indeed, given the essential nature of SAM for a plethora of cellular transmethylation reactions including, but not limited to, epigenetic regulation [43], and the potential inhibitory action of SAH on cellular methyltransferases [44], the metabolically catastrophic potential of dysregulating GliT-mediated control of GT/BmGT biosynthesis is only just becoming apparent. Further systems impacts of interfering with GT biosynthesis are emerging (i.e., aberrant zinc homeostasis [22]) and, surprisingly, we have shown that the biosynthesis of apparently unrelated natural products, like the powerful antioxidant ergothioneine, is influenced either by GT [45,46], or specific reactions within its biosynthetic pathway [47]. So, the activity of GT against fungi and animal cells is revealing new interactions within biological systems, including zinc homeostasis [22] as has been discussed elsewhere [16], and now merits consideration to identify new drug targets in bacteria (Figure 2A).

Experimentally, quantitative proteomic analysis, and parallel RNAseq [26], allied to intracellular metabolite quantification, have uncovered hitherto unforeseen systems interactions between GT/BmGT biosynthesis, self-resistance, altered sulphur metabolism and zinc homeostasis in *A. fumigatus* [48]. In combination, this dissection of GT biosynthesis and self-resistance has led to the discovery of new aspects of fungal systems biology [16], and now underpins the concept of GT deployment to identify essential bacterial systems (e.g., those responsible for cellular metal ion homeostasis and methylation systems) and bacterial drug targets, the inhibition of which can lead to circumventing or overcoming AMR [49] (Figure 1). Given that GT also exhibits significant antibacterial activity [50,51], of unknown mechanistic etiology but now hypothesised to involve interference with zinc homeostasis/metalloenzyme function and methylation, future molecular dissection of GT-mediated bacterial growth inhibition may reveal new avenues to identify new antibiotic targets, rescue antibiotic sensitivity and overcome AMR, in bacteria (Figure 1).

#### GT and holomycin equivalence

Quantitative proteomic approaches have been extensively used to dissect GT resistance and functionality in *A. fumigatus*, as well as its impact on cellular homeostasis, in fungi, and it has been revealed that DTG is a zinc chelator, which positions DTG as a Zn^2+^-metalloenzyme inhibitor [16,22,34]. In *A. fumigatus*, GT oxidoreductase GliT catalyses resistance to the reactive form of GT, DTG [30,33]. In addition, it has been demonstrated that GT *bis*-thiomethylation is mediated by *bis*-thiomethyltransferase GtmA which requires SAM as a co-substrate, to effect attenuation of GT biosynthesis [34]. While it is explicitly not conceptualised that GT can be exploited as an antibiotic, given that bacteria are sensitive to GT, it is essential to consider innovative exploitation of GT as a *Pathfinder* to identify new antibiotic targets and overcome AMR.

Thus, historical and emerging literature [19,50,52], the ground-breaking finding that DTG can eject Zn^2+^ from metalloenzymes [22], plus emerging data and the functional equivalence between GT and holomycin, provides a convincing and urgent rationale for investigating the aforementioned bacterial sensitivity to GT as a novel and highly promising direction in the fight against AMR (Figure 2). Studies on GT and bacterial holomycin biosynthesis and self-resistance, in *A. fumigatus* and *Streptomyces clavuligerus*, respectively, have occurred in parallel since 2010, with concepts reciprocally contributing to the respective field. For instance, the seminal discovery of *gliT*-mediated self-resistance against GT [33] was mirrored in the observation of *hlmI*-mediated self-protection against holomycin in *S. clavuligerus* [37]. The observation of *bis*-thiomethylated holomycin derivatives in *S. clavuligerus* ∆*hlmI*, which was posited as a back-up self-resistance mechanism [53], preceded the discovery of GtmA-mediated gliotoxin *bis*-thiomethyltransferase in *A. fumigatus*. In the latter species, as noted above it has been demonstrated that GtmA primarily serves a negative regulatory biosynthetic, as opposed to self-protection, function [34]. The enzyme which mediates *bis*-thiomethylation of dithiol holomycin (Figure 2C) remains to be discovered, thereby limiting holomycin use as a Pathfinder.

Holomycin biosynthesis is encoded by the *hom* gene cluster in the pathogen, *Yersinia ruckeri* [54]. However, the *Y*. *ruckeri hom* cluster lacks the *hlmI* self-resistance gene found in *S. clavuligerus*, and instead encodes a SAM-dependant RNA methyltransferase, termed *hom12*. Absence of *hom12* results in acquisition of holomycin sensitivity in *Y*. *ruckeri*, and Hom12 has been demonstrated to directly methylate RNA, which has resulted in the proposal that Hom12-mediated-RNA methylation is a novel self-resistance mechanism to facilitate holomycin production in bacteria lacking *hlmI* [54]. As yet, the exact nature of the RNA species which is methylated, and position(s) of methylation are unknown. Aminoglycoside, macrolide and lincosamide antibiotics inhibit bacterial protein synthesis by targeting RNA. Methylation also directly inhibits antibiotic function in bacteria [55]. Relevantly, the primary means of bacterial resistance to macrolide antibiotics is by post-transcriptional methylation of the 23S bacterial ribosomal RNA [56]. Moreover, RNA methylation may be deployed in certain antibiotic-producing bacteria to alter ribosome structure (via rRNA modification), thereby reducing the affinity for, and consequent activity of, the cognate antibiotic. Nascent self-protection and resistance mechanisms against holomycin clearly illuminate a biochemical link between antibiotic resistance and methylation. Consequently, it is evident that interference with cellular methylation in bacteria could disrupt the capacity of bacteria to exploit RNA methylation as an antibiotic resistance strategy. Thus, combinatorial chemoenzymatic approaches of using both GT exposure and *in vivo* GtmA activity to deplete cellular SAM levels in bacterial *spp.*, to assess if altered substrate availability for SAM-dependant RNA methyltransferases could prevent the development of resistance mechanisms mediated by RNA methylation are merited.

#### GT and zinc

**Figure 3:**
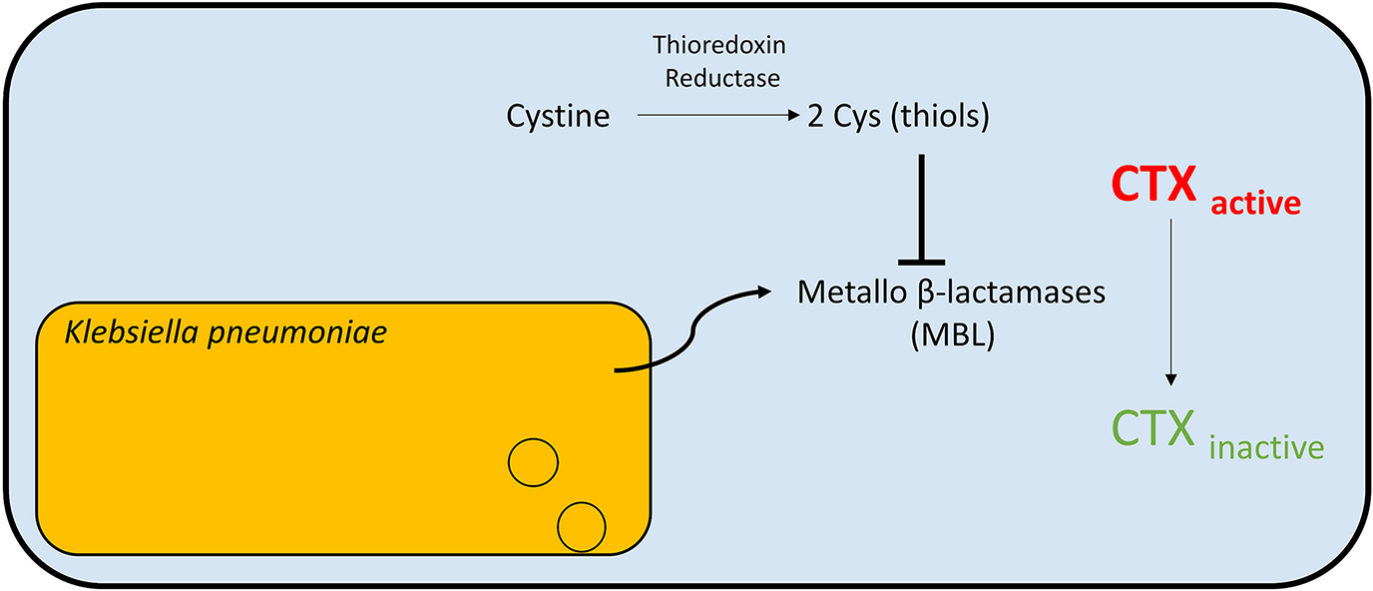
Thiol compounds inhibit MBL and re-sensitise bacteria to the antibiotic Zinc-dependent metallo β-lactamases (MBL) are secreted by *K. pneumoniae* and hydrolyse cefotaxime antibiotic (CTX), which facilitates bacterial resistance to the antibiotic. Thiol compounds in culture media or urine (e.g., endogenous L-Cys or that produced by thioredoxin reductase activity on L-cystine) can either bind free zinc or inhibit MBL via zinc ejection and prevent MBL degradation of CTX. This re-sensitizes the bacteria to the antibiotic [65].

GT contains an intramolecular disulphide bridge and can undergo intracellular chemical reduction, mediated by GSH, to DTG [38]. While GSH is not produced by all bacterial spp. that does not rule out GT reduction and thiol depletion as a means of GT-induced bacterial growth inhibition, since there are other naturally occurring thiols with similar functions. These include mycothiol (MSH), L-Cys, H_2_S, and coenzyme A [57], moreover, the primary thiol depends on the species in question, with GSH more evident in actinomycetes while non-actinomycetes spp. primarily produce MSH. Tris(2-carboxyethyl)phosphine (TCEP) and other reductants facilitate *in vitro* DTG formation from GT [58]. GT is produced by *A. fumigatus*, in what recent studies suggest is in a zinc-dependent manner [21,22,24,59]. DTG is a biosynthetic intermediate in the GT biosynthetic pathway and likely formed by GSH-mediated reduction of GT in *A. fumigatus*, however its presence is detrimental and two enzymes and a membrane transporter (GliT, GtmA and GliA) are present to effect its dismutation and secretion. Indeed, GT and other ETPs not only inhibit fungal growth but have been shown to inhibit the growth of multiple bacterial species [60,61] (Table 1) and also possibly prevent biofilm formation [51]. Mainly studied in fungal settings [20,45,62,63], there is limited research into the GT mechanism of action on bacterial species. Indeed, apart from a publication inferring interference with GSH levels, no mechanistic details have been forthcoming about how GT inhibits bacterial growth [64]. Interestingly, antibiotic (cefotaxime; CTX) resistance of *K. pneumoniae* has been overcome by thiol-mediated inhibition of zinc metallo-β-lactamase activity [65]. Specifically, L-Cys was identified as an endogenous zinc chelator, which rescued metallo-β-lactamase-secreting *K. pneumoniae* to CTX (Figure 3).

Since our demonstration that GT-mediated growth inhibition of specific *A. fumigatus* mutants is substantially due to zinc chelation, depletion and possibly metalloenzyme inhibition, several publications have emerged focussing on the GT/Zn axis in *A. fumigatus*. However, here we go further and based on literature observations [11,22,66] and unpublished preliminary data, hypothesise that the potent zinc-chelating ability of GT, or more correctly, DTG (i) has the potential to inhibit the growth of other species, especially bacteria and (ii) reveal critical zinc-dependent metalloenzymes or pathways essential for microbial growth. While we are *not* proposing GT/DTG directly as a potential antimicrobial agent for clinical use, the deployment of GT/DTG as a Pathfinder to highlight essential zinc-dependent functionalities in bacterial spp. merits urgent attention, particularly in the ESKAPE group of organisms and the continued emergence of antibiotic resistance.

### Antimicrobial effect of GT on Gram positive and negative bacteria

The antibiotic effects of GT are well documented against *S. aureus* [50,51,67]. The MIC of GT has been determined to range between 0.23 and 4 µg/ml for *S. aureus*, including ATCC29213 and unknown strains of *S. aureus* [49,68–70] (Table 1). GT was also found to be an effective growth inhibitor of MRSA strains with an MIC range of 0.32–2 µg/ml, and multi-drug resistant *S. aureus* (MDRSA) with an MIC 1 µg/ml [49,50,68,69,71] (Table 1). In both broth dilution and agar-plate assays, GT inhibited MRSA ATCC700699 (MIC: 2 µg/ml) [49], a strain that is also classified as vancomycin intermediate-resistant (VISA). While most *Staphylococcus* spp. work has focussed on *S. aureus*, GT (2.5 µg/ml) has been shown to inhibit the growth of *Staphylococcus epidermidis* on agar [70]. *Streptococcus* spp. have been shown to be susceptible to low levels of GT. The GT MIC for *Streptococcus* piscine pathogens was observed to range from 0.08 to 0.64 µg/ml, using the McFarland method [71] (Table 1). This accords with earlier work by Johnson et al. who found that GT (1 µg/ml) had a bacteriostatic effect on a member of the viridans group streptococci [70]. Studies reporting the effect of GT on clinically relevant *Streptococcus spp*. is limited and deserves further investigation. *Lactococcus garvieae* FP5245 exhibited a GT MIC of 0.32 µg/ml, as did MDR *Enterococcus faecium* 5207 [71]. Vancomycin-resistant *Enterococcus faecalis* V4932 was shown to be susceptible to culture filtrates containing GT, even when the filtrates were quite dilute (1:512) [50]. *Bacillus subtilis* growth is also inhibited by GT although the MIC has not yet been established [67]. GT (3.0 µg/ml) has been shown to have a bacteriostatic effect on *B. subtilis*, leading to increased lag phase of the species to 300 min ± 20 [72]. *M. tuberculosis* H37Rv was found to be particularly sensitive to GT with an IC90 of 0.05 µg/ml [73]. This sensitivity to GT has been reflected in several other strains of *M. tuberculosis*, with MIC values ranging from 0.006 to 0.06 µg/ml [19,74]. GT (0.25 µg/ml for 48 h) also significantly inhibited biofilm formation of *S. aureus* (*P*=0.024) [51]. Strangely, in this study there was a larger percentage biofilm remaining after 48 h when 0.33 µg/ml GT was used when compared to 0.25 µg/ml GT, which had the highest inhibitory effect of the concentrations tested.

**Table 1:** The effect of GT on various bacterial species

Bacterial species/strain	GT MIC (µg/ml)	Comments
***Gram (+)***		
*Staphylococcus aureus*		
ATCC29213 [68]	4	
ATCC29213 [49]	4	
ATCC29213 [50]	ND	GT-containing extracts
Unknown strain [69]	1	
Unknown strain [70]	1	
Unknown strain [70]	0.23	
Unknown strain [51]	ND	Biofilm: Inhibition at 0.25 µg/ml
Unknown strain [67]	ND	Zone of inhibition (27 mm) observed on agar
*Antibiotic resistant S. aureus*		
MRSA 2.08 [50]	ND	GT-containing extracts
MRSA R3708 [68]	1	
MRSA ATCC700699 (VISA) [49]	2	
MRSA 3089 [71]	0.32	
MRSA [69]	1	
MDRSA [69]	1	
*Staphylococcus epidermidis* [70]	2.5	
*Streptococcus iniae FP5228* [71]	0.64	
*S. iniae* FP3187 [71]	0.16	
*Streptococcus parauberis* FP3287 [71]	0.08	
*S. parauberis* SPOF3K [71]	0.16	
*S. parauberis* KSP28 [71]	0.16	
*Viridans group streptococcus* [70]	1	
*Lactococcus garvieae* FP5245 [71]	0.32	
*MDR Enterococcus faecium* 5207 [71]	0.32	
*VRE Enterococcus faecalis V4932* [50]	ND	GT-containing extracts
*Bacillus subtilis* [67]	ND	Zone of inhibition (23 mm) observed on agar
*B. subtilis* [72]	ND	Increased lag phase on GT (3 µg/ml)
*Mycobacterium tuberculosis* [73]	ND	IC_90_: 0.05 µg/ml
*M. tuberculosis* [74]	< 0.03	MIC50: < 0.03 µg/ml
*M. tuberculosis* (several strains) [19]	0.006-0.06	
***Gram (-)***		
*Escherichia coli* [50]	ND	GT-containing extracts
*E. coli* [67]	ND	Zone of inhibition (31 mm) observed on agar
*E. coli* [70]	10	
*E. coli* ESBL [50]	ND	GT-containing extracts
*MDR E. coli* 1137 [71]	10	
*S. typhimurium* (LT2) [64]	ND	GT (3 µg/ml) inhibited growth of 1.5 × 10^4^ cells per 5 ml
*S. typhimurium* 1181 deep rough mutant [64]	ND	GT (1.5 µg/ml) inhibited growth of 2.0 × 10^6^ cells per 5 ml
*Salmonella schottmuelleri* [70]	>10	
*MDR Salmonella typhimurium* 8173 [71]	10	
*Salmonella Paratyphi* [70]	5	
*Pseudomonas aeruginosa* [51]	ND	Biofilm inhibition at 0.13 µg/ml
*Pseudomonas fluorescens* [67]	ND	Zone of inhibition (26 mm) observed on agar
*Klebsiella pneumoniae* [50]	ND	GT-containing extracts
*Bordetella pertussis* [70]	2.5	
*Moraxella catarrhalis* [70]	0.2	
*Proteus sp*. (MTCC No. 426) [67]	ND	Zone of inhibition (29 mm) observed on agar
*Vibrio ichthyoenteri* 0917-1 [71]	10	
*V. ichthyoenteri* FP8487 [71]	20	
*MDR Vibrio parahaemolyticus* 7001 [71]	>40	
*MDR Enterobacter cloacae* P990252 [71]	>40	
*Acinetobacter baumannii* [51]	ND	Biofilm inhibition at 0.13 µg/ml
*Burkholderia multivorans* [51]	ND	Biofilm inhibition at 0.13 µg/ml
*Haemophilus influenzae* [51]	ND	Biofilm inhibition at 0.065 µg/ml

For many Gram negative species, higher dosages were required to effect growth inhibition compared to Gram positive bacteria [50,51,67,70,71] (Table 1). *E. coli*, including MDR strains, exhibit some sensitivity to GT (MIC: 10 µg/ml) [50,67,70,71]. *Salmonella* spp. including MDR *Salmonella typhimurium* 8173 are also sensitive to GT (MIC: 5–10 µg/ml) [64,70,71]. In contrast, MDR *Enterobacter cloacae* P990252 and MDR *Vibrio parahaemolyticus* 7001 are considered resistant to GT (MIC: >40 µg/ml) [71]. Thus, while GT does possess broad-spectrum activity against Gram negative spp., it is less effective than against Gram positive strains, with higher concentrations required to achieve the same growth inhibitory effect.

GT has also been shown to inhibit biofilm formation in mixed pathogen/*A. fumigatus* cultures where it was active against *A. baumannii*, *Burkholderia multivorans*, *Haemophilus influenzae*, and *P. aeruginosa* where the highest reduction in biofilm formation occurred at GT 0.25, 0.33, 0.06, and 0.2 µg/ml, respectively [51] (Table 1). In co-cultures, it was noted that *A. fumigatus* inhibited *P. aeruginosa* growth due to the production of GT, which was increased in the presence of *P. aeruginosa* [75]. This inhibition could be rescued by the production of phenazines, redox-active compounds which are toxic to the competing organism [76], by *P. aeruginosa*. If the phenazine-producing *P. aeruginosa* strain did not produce the phenazine, pyocyanin, which is responsible for upregulation of genes that function in transport and possibly in redox control, as well as down-regulating genes involved in ferric iron acquisition [77], conidia production in *A. fumigatus* was reduced. In species which do produce the phenazine pyocyanin the opposite occurs, causing enhanced conidia production. Jones and Hancock studied a deep rough mutant of *Salmonella typhimurium*, characterised by a reduction in outer membrane polysaccharide content, and wild-type *S. typhimurium* (LT2) growth in the presence of GT at 24 h [64]. The deep rough mutant was found to be more sensitive to GT than the wild-type *S. typhimurium*, indicating the outer membrane provides a protective role against GT [64].

Of further relevance is that it has been independently shown that GT acts synergistically with vancomycin, fusidic acid and linezolid, to inhibit *S. aureus* growth [49], an observation which suggests differential mechanistic actions and strongly underpins our rationale for further investigating the GT/DTG couple. These authors did not suggest a metal-dependent basis of GT-mediated inhibition. However, although GT/DTG augments the vancomycin-mediated inhibition of bacterial growth, the impact of zinc addition, or known zinc chelators, after observed combinatorial inhibition is unknown. If the zinc chelator N,N,N′,N′-*tetrakis*-(2-pyridylmethyl)ethylenediamine (TPEN) does not synergise with vancomycin to cause increased growth inhibition, this would further underpin the uniqueness of GT/DTG and add urgency to establishing its inhibitory action against bacteria.

### GT as a probe to investigate the integratory role of zinc in bacteria

Not only is zinc a component of up to 6% of all proteins in prokaryotes [78], but several ribosomal proteins contain zinc-binding domains [79]. In seminal work, zinc has been proposed to be an integrator of metabolism whereby zinc-dependent enzymes, especially those involved in folic acid biosynthesis, sense free zinc availability and somehow control zinc chaperone activity, in addition to adjusting the zinc content of ribosomes [80]. Moreover, based on its recently observed zinc-chelating ability [59], Danchin astutely proposed that GT could be used to investigate the biological functions of zinc in cells. We contend that this completely independent verification and validation of our evidence-based hypothesis that the zinc-chelating properties of GT, or more precisely, DTG [22], have the potential to reveal new insights into the central role of zinc in biological systems, especially bacterial systems, merits serious investigation. Indeed, the significant perturbation of eukaryotic methyl (one-carbon)/methionine cycle due to dysregulated thiomethylation via GT thiomethyltransferase GtmA activity in *A. fumigatus* [41,81], especially following deletion of GT oxidoreductase GliT, is functionally equivalent to Danchin’s hypothesis which highlights the overlapping importance of zinc in one-carbon metabolism, SAM synthesis and translation [80,82]. Furthermore, the reversal of severe GT-mediated growth inhibition in the double-mutant *A. fumigatus* Δ*gliT*Δ*gtmA* by zinc addition [22,42], further underpins this nascent gliotoxin-zinc nexus as a strategy for investigating the importance of zinc homeostasis, and the essential role of zinc, in prokaryotes naturally lacking the DTG-dissipating enzymes, GliT and GtmA.

To exploit GT as a Pathfinder the following strategies could be explored: (1) Investigate if GT has global or targeted effects on zinc-metalloenzymes in bacteria; (2) Establish the relative importance of GT ability to chelate free, or eject protein-bound, Zn^2+^; (3) Elucidate the relative contribution of GT as a chelator or ionophore; and (4) Explore, in detail, the synergistic antimicrobial activity of GT with known or emerging antimicrobials (Figure 2). Resultant identification of important GT-sensitive enzymes as virulence contributors could then be validated by deletion studies, and then disrupted by target-specific inhibitors, as antimicrobials, to circumvent the eukaryotic cytotoxicity of GT.

## Concluding remarks

The GT/DTG couple has significant potential to identify cryptic bacterial intracellular systems as potential drug targets and to sensitise bacteria against pre-existing antibiotics (Figures 1-3). Immediate work is required to mechanistically investigate how this couple can inhibit bacterial growth and exploit this potential to overcome AMR.

## Summary

- Antimicrobial resistance is a major global problem and new antimicrobials are urgently required.
- Thiol-containing compounds and ionophores have potential to provide new directions to overcome antimicrobial resistance.
- The reduced form of disulphide-containing, fungal metabolite, gliotoxin (GT) (dithiol gliotoxin [DTG]) is a zinc chelator, and zinc homeostasis has been shown to be essential for bacterial virulence.
- GT/DTG can inhibit bacterial growth and augment antibiotic activity by unknown mechanisms.
- Zinc may be an integrator of metabolism in bacteria, therefore interference with zinc homeostasis, via GT/DTG, has potential to identify new drug targets.

## Abbreviations

1C: one carbon
AMR: antimicrobial resistance
BmGT: bisdethiobis(methylthio)gliotoxin
CTX: cefotaxime
DTG: dithiol gliotoxin
DTP: dithiolopyrrolone
ETP: epipolythiodioxopiperazine
FDA: Food and Drug Administration
GSH: glutathione
GT: gliotoxin
L-Cys: L-cysteine
MDR: multi-drug resistant
MDRSA: multidrug-resistant *Staphylococcus aureus*
MIC: minimum inhibitory concentration
MRSA: methicillin-resistant *Staphylococcus aureus*
MSH: mycothiol
ROS: reactive oxygen species
SAH: *S*-adenosyl homocysteine
SAM: S-adenosyl methionine
TCEP: Tris(2-carboxyethyl)phosphine
TPEN: N,N,N′,N′-*tetrakis*-(2-pyridylmethyl)ethylenediamine
VISA: vancomycin intermediate *Staphylococcus aureus*
VRE: vancomycin-resistant enterococci
